# Supplementary material for: Development of Drug-Induced Gene Expression Ranking Analysis (DIGERA) and Its Application to Virtual Screening for Poly (ADP-Ribose) Polymerase 1 Inhibitor
Source: Int J Mol Sci. 2024 Dec 30;26(1):224. doi: 10.3390/ijms26010224 (PMC11720423; doi:10.3390/ijms26010224)
Supplement: Supplementary file 1 [file ijms-26-00224-s001.zip › ijms-3349101-supplementary.pdf]

Supporting Information for

Development of Drug-Induced Gene Expression Ranking Analysis (DIGERA) and Its Application to Virtual Screening for Poly (ADP-Ribose) Polymerase 1 Inhibitor

Hyein Cho[1,†], Kyoung Tai No[1,2,3,*], and Hocheol Lim[2,†,*]

[1] The Interdisciplinary Graduate Program in Integrative Biotechnology & Translational Medicine, Yonsei University, Incheon, Republic of Korea

[2] Bioinformatics and Molecular Design Research Center (BMDRC), Incheon, Republic of Korea

[3] Baobab AiBIO Co., Ltd., Incheon, Republic of Korea

[†] These authors contributed equally to this work.

[*] Corresponding authors: Hocheol Lim (ihc0213@yonsei.ac.kr) and Kyoung Tai No (ktno@yonsei.ac.kr)

The supporting information for 'Development of Drug-Induced Gene Expression Ranking Analysis (DIGERA) and Its Application to Virtual Screening for Poly (ADP-Ribose) Polymerase 1 Inhibitor' includes Figures S1-S3 for heatmaps of performance metrics in up/down-regulated gene expression ranking prediction, Table S1 for hyperparameter tuning procedure, Table S2 for optimal hyperparameters in gene expression ranking prediction models, Table S3-S5 for performance metrics of the up, down, and up/down-regulated gene expression ranking prediction in a single cell line, Table S6-S7 for performance metrics of the up and down-regulated expression ranking prediction in multiple cell lines, Tabls S8 for optimal hyperparameters in QSAR models for PARP1, and Table S9 for performance metrics of QSAR models for PARP1.

In this study, there are many abbreviations as follows. CIGER, Chemical-Induced Gene Expression Ranking; CRISPR, Clustered Regularly Interspaced Short Palindromic Repeats; DIGERA, Drug-Induced Gene Expression Ranking Analysis; ECFP, Extended-Connectivity Fingerprints; FCFP, Function-Class Fingerprints; FNN, Feedforward Neural Networks; GA, Genetic Algorithms; GCN, Graph Convolutional Network; HIA, Human Intestinal Absorption; Lasso, Least Absolute Shrinkage Selector Operator; LGBM, Light Gradient Boosting Machine; LINCS, Library Of Integrated Network-Based Cellular Signatures; LogP, Water-Octanol Partition Coefficient; LSTM, Long-Short-Term Memory; MACCS, Molecular Access System; MCC, Matthews Correlation Coefficient; MFBERT, Molecular Fingerprints Through Bidirectional Encoder Representations From Transformers; NLP, Natural Language Processing; PARP1, Poly (ADP-Ribose) Polymerase 1; PCA, Principal Component Analysis; PCFP, PubChem Fingerprint; QSAR, Quantitative Structure-Activity Relationship; QSPR, Quantitative Structure-Property Relationship; RAscore, Retrosynthetic Accessibility Score; RFC, Random Forest Classifier; RFR, Random Forest Regressors; RMSD, Root-Mean-Square Deviation; RNN, Recurrent Neural Network; SAGE, Scoring-Assisted Generative Exploration; SMILES, Simplified Molecular-Input Line-Entry System; XGB, Extreme Gradient Boosting.

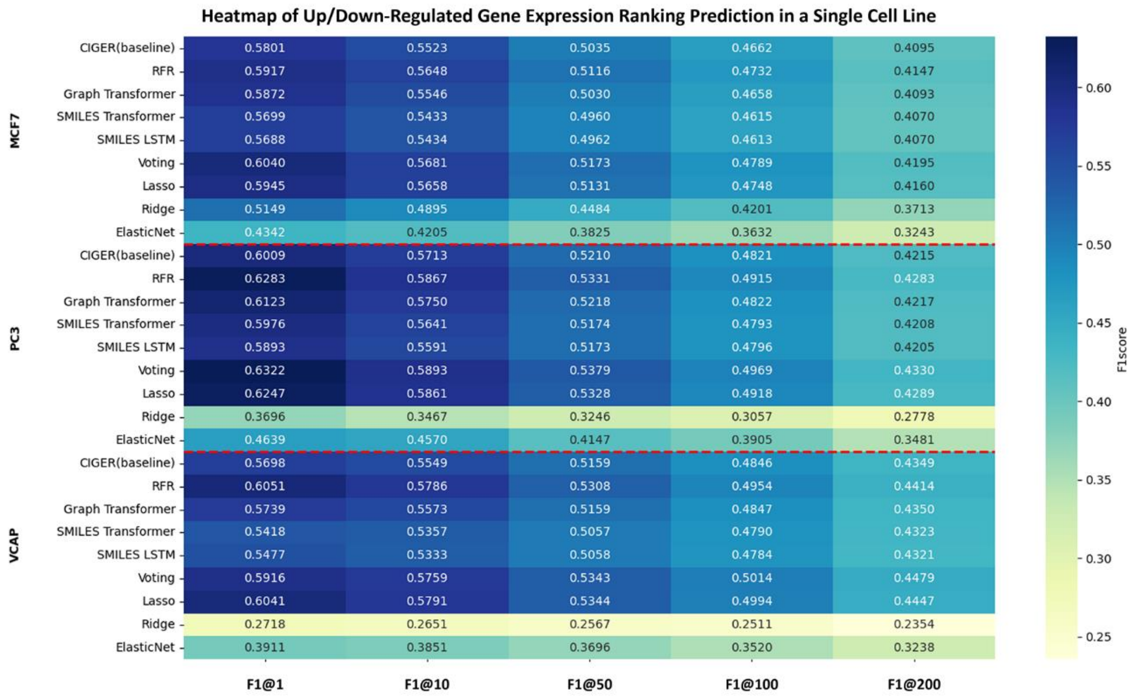

Figure S1. Heatmaps of Performance Metrics in Up/Down-Regulated Gene Expression Ranking Prediction in a Single Cell Line (MCF7, PC3, and VCAP)

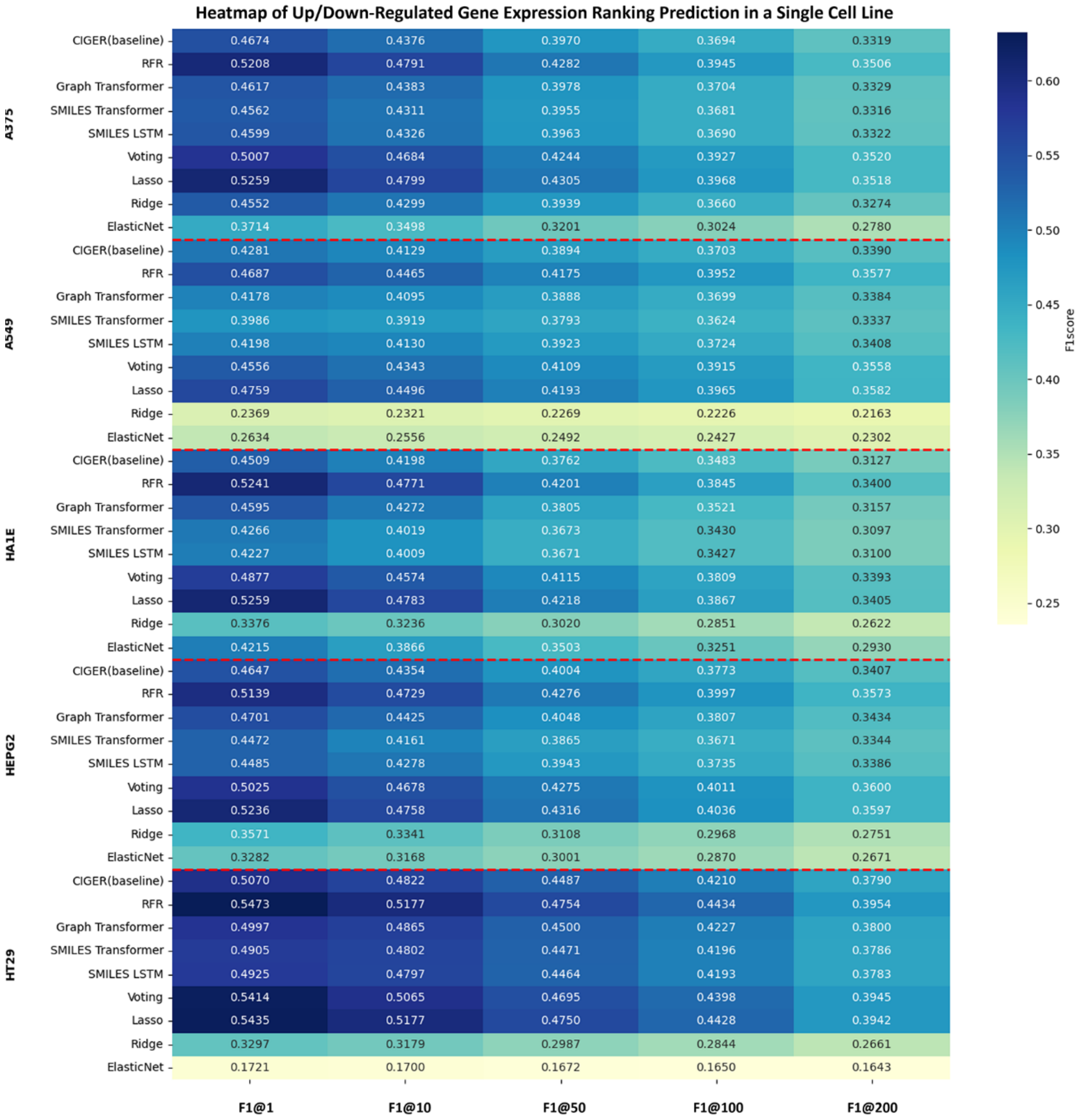

Figure S2. Heatmaps of Performance Metrics in Up/Down-Regulated Gene Expression Ranking Prediction in a Single Cell Line (A375, A549, HA1E, HEPG2, and HT29)

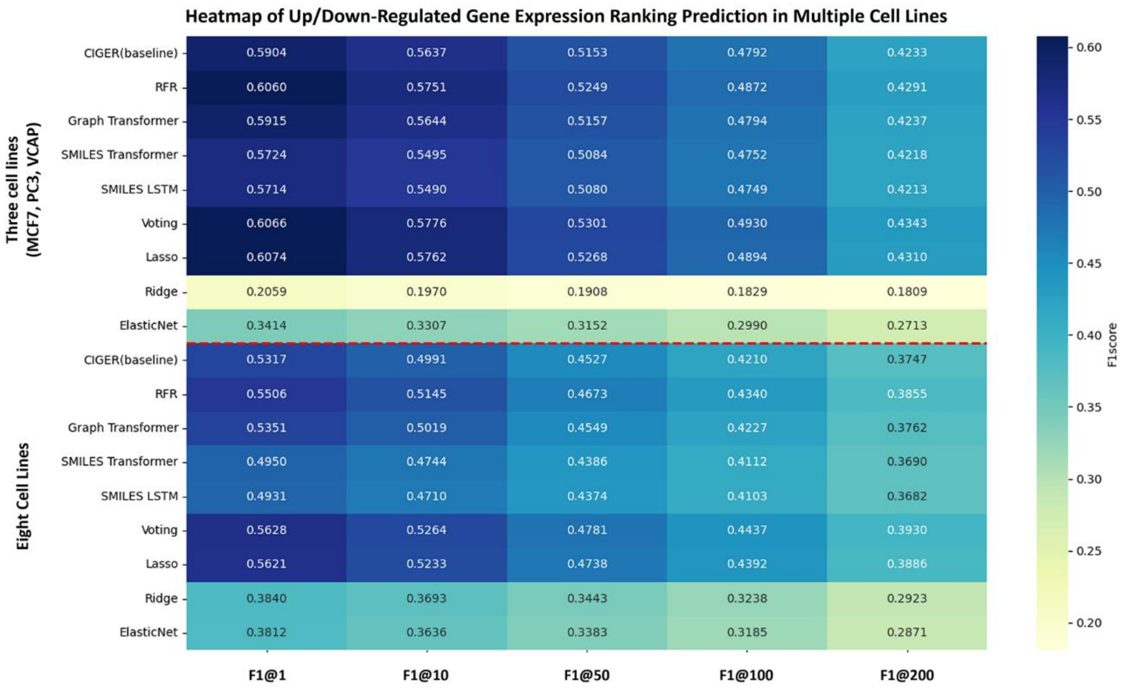

Figure S3. Heatmaps of Performance Metrics in Up/Down-Regulated Gene Expression Ranking Prediction in Multiple Cell Lines

Table S1. Parameters Used in the Hyperparameter Tuning Procedure

Class	Method	Tuning parameters	Fixed parameters
Gene Expression Ranking	CIGER (baseline)		layer = 1, head = 1<br>loss = listwise_rankcosine
	RFR	n_estimators = 50, 100, 500, 1000, 1500, 2000, 2500, 3000<br>max_depth = 15, 20, 25<br>max_features = 'auto'. 'sqrt'	loss = mse
	Graph Transformer	layer = 1, 2, 3, 4, 5<br>head = 1, 2, 4, 8<br>loss = 'pointwise_mse', 'listwise_rankcosine'	
	SMILES Transformer	layer = 1, 2, 3, 4<br>dmodel = 64, 128, 256, 512, 1024<br>hidden_unit = 128, 256, 512, 1024<br>head = 1, 2, 4, 8<br>loss = 'pointwise_mse', 'listwise_rankcosine'	
	SMILES LSTM	layer = 1, 2, 3, 4<br>dmodel = 64, 128, 256, 512, 1024<br>hidden_unit = 128, 256, 512, 1024<br>loss = 'pointwise_mse', 'listwise_rankcosine'	
	Lasso (Ensemble)<br>Ridge (Ensemble)<br>ElasticNet (Ensemble)		c = 0.2<br>l = 0.01
PARP1	LGBM	boosting_type = 'gbdt', 'dart'<br>n_estimators = 50, 100, 500, 1000, 1500, 2000, 2500, 3000<br>learning_rate = 0.01, 0.05, 0.1	class_weight = 'balanced'
	RFC	n_estimators = 50, 100, 500, 1000, 1500, 2000, 2500, 3000<br>max_depth = 10, 20, 30	class_weight = 'balanced'
	XGB	booster = 'gbtree', 'dart'<br>n_estimators = 50, 100, 500, 1000, 1500, 2000, 2500, 3000<br>max_depth = 10, 20, 30<br>learning_rate = 0.01, 0.05, 0.1	class weight is balanced through sample_weight

Table S2. Optimal Hyperparameters of Gene Expression Ranking Prediction Models

Model	Optimal Parameter
CIGER (baseline)	{'layer': 1, 'head': 1, 'loss': 'listwise_rankcosine'}
RFR	{'n_estimators': 2500, 'max_depth': 20, 'max_features': 'auto'}
Graph Transformer	{'layer': 2, 'head': 1, 'loss': 'pointwise_mse'}
SMILES Transformer	{'layer': 1, 'dmodel': 64, 'hidden_unit': 1024, 'head': 1, 'loss': 'listwise_rankcosine'}
SMILES LSTM	{'layer': 1, 'dmodel': 512, 'hidden_unit': 128, 'loss': 'listwise_rankcosine'}
Lasso (Ensemble)	{'c': 0.2, 'l': 0.01}
Ridge (Ensemble)	{'c': 0.2, 'l': 0.01}
ElasticNet (Ensemble)	{'c': 0.2, 'l': 0.01}

Table S3. Performance Metrics of Up-Regulated Gene Expression Ranking Prediction in a Single Cell Line (continue)

Class	Cell line	Model	F1@1	F1@10	F1@50	F1@100	F1@200
Up	MCF7	CIGER (baseline)	0.5470 ± 0.0170	0.5053 ± 0.0040	0.4529 ± 0.0059	0.4163 ± 0.0049	0.3712 ± 0.0034
		RFR	0.5650 ± 0.0037	0.5247 ± 0.0091	0.4624 ± 0.0084	0.4237 ± 0.0058	0.3765 ± 0.0042
		Graph Transformer	0.5616 ± 0.0119	0.5098 ± 0.0050	0.4525 ± 0.0068	0.4157 ± 0.0055	0.3709 ± 0.0042
		SMILES Transformer	0.5372 ± 0.0105	0.4918 ± 0.0069	0.4384 ± 0.0060	0.4053 ± 0.0055	0.3644 ± 0.0041
		SMILES LSTM	0.5318 ± 0.0175	0.4940 ± 0.0070	0.4385 ± 0.0073	0.4055 ± 0.0057	0.3647 ± 0.0046
		Voting (Ensemble)	**0.5772 ± 0.0116**	**0.5294 ± 0.0056**	**0.4727 ± 0.0074**	**0.4341 ± 0.0057**	**0.3863 ± 0.0041**
		Lasso (Ensemble)	0.5657 ± 0.0058	0.5248 ± 0.0071	0.4638 ± 0.0077	0.4252 ± 0.0059	0.3780 ± 0.0042
		Ridge (Ensemble)	0.4934 ± 0.0694	0.4561 ± 0.0645	0.4116 ± 0.0514	0.3824 ± 0.0438	0.3405 ± 0.0374
		ElasticNet (Ensemble)	0.4410 ± 0.1447	0.4158 ± 0.1151	0.3621 ± 0.1105	0.3409 ± 0.0914	0.3034 ± 0.0821
	PC3	CIGER (baseline)	0.5617 ± 0.0248	0.5201 ± 0.0135	0.4624 ± 0.0101	0.4254 ± 0.0087	0.3785 ± 0.0065
		RFR	0.5922 ± 0.0281	0.5384 ± 0.0174	0.4761 ± 0.0120	0.4352 ± 0.0107	0.3854 ± 0.0077
		Graph Transformer	0.5820 ± 0.0158	0.5229 ± 0.0106	0.4640 ± 0.0095	0.4259 ± 0.0082	0.3792 ± 0.0062
		SMILES Transformer	0.5571 ± 0.0282	0.5061 ± 0.0147	0.4545 ± 0.0102	0.4186 ± 0.0101	0.3749 ± 0.0071
		SMILES LSTM	0.5453 ± 0.0148	0.5023 ± 0.0151	0.4543 ± 0.0097	0.4191 ± 0.0100	0.3743 ± 0.0075
		Voting (Ensemble)	**0.6033 ± 0.0191**	**0.5449 ± 0.0111**	**0.4866 ± 0.0107**	**0.4473 ± 0.0089**	**0.3962 ± 0.0067**
		Lasso (Ensemble)	0.5881 ± 0.0200	0.5383 ± 0.0151	0.4751 ± 0.0116	0.4356 ± 0.0102	0.3862 ± 0.0075
		Ridge (Ensemble)	0.3471 ± 0.2202	0.3134 ± 0.2030	0.2898 ± 0.1811	0.2757 ± 0.1563	0.2550 ± 0.1255
		ElasticNet (Ensemble)	0.4352 ± 0.2002	0.4338 ± 0.1267	0.3883 ± 0.1064	0.3630 ± 0.0872	0.3221 ± 0.0781
	VCAP	CIGER (baseline)	0.5989 ± 0.0107	0.5609 ± 0.0075	0.5077 ± 0.0070	0.4706 ± 0.0060	0.4200 ± 0.0039
		RFR	**0.6323 ± 0.0044**	**0.5938 ± 0.0092**	0.5293 ± 0.0066	0.4867 ± 0.0058	0.4292 ± 0.0042
		Graph Transformer	0.5974 ± 0.0120	0.5612 ± 0.0055	0.5071 ± 0.0050	0.4699 ± 0.0048	0.4194 ± 0.0033
		SMILES Transformer	0.5497 ± 0.0122	0.5274 ± 0.0090	0.4871 ± 0.0059	0.4580 ± 0.0047	0.4132 ± 0.0043
		SMILES LSTM	0.5539 ± 0.0125	0.5254 ± 0.0088	0.4879 ± 0.0067	0.4575 ± 0.0058	0.4135 ± 0.0047
		Voting (Ensemble)	0.6198 ± 0.0110	0.5869 ± 0.0089	**0.5313 ± 0.0078**	**0.4924 ± 0.0055**	**0.4372 ± 0.0042**
		Lasso (Ensemble)	0.6300 ± 0.0105	0.5881 ± 0.0108	0.5284 ± 0.0079	0.4869 ± 0.0061	0.4303 ± 0.0044
		Ridge (Ensemble)	0.2845 ± 0.2474	0.2703 ± 0.2259	0.2542 ± 0.2042	0.2464 ± 0.1850	0.2301 ± 0.1527
		ElasticNet (Ensemble)	0.4171 ± 0.2342	0.3912 ± 0.2187	0.3628 ± 0.1936	0.3415 ± 0.1754	0.3126 ± 0.1498

Table S3. Performance Metrics of Up-Regulated Gene Expression Ranking Prediction in a Single Cell Line (continue)

Class	Cell line	Model	F1@1	F1@10	F1@50	F1@100	F1@200
Up	A375	CIGER (baseline)	0.4559 ± 0.0121	0.4145 ± 0.0078	0.3712 ± 0.0056	0.3468 ± 0.0050	0.3159 ± 0.0033
		RFR	0.5238 ± 0.0202	0.4668 ± 0.0090	0.4067 ± 0.0042	0.3740 ± 0.0022	0.3361 ± 0.0012
		Graph Transformer	0.4411 ± 0.0275	0.4121 ± 0.0082	0.3701 ± 0.0044	0.3463 ± 0.0028	0.3162 ± 0.0024
		SMILES Transformer	0.4429 ± 0.0231	0.3969 ± 0.0066	0.3624 ± 0.0025	0.3391 ± 0.0028	0.3116 ± 0.0030
		SMILES LSTM	0.4459 ± 0.0227	0.4034 ± 0.0064	0.3637 ± 0.0066	0.3407 ± 0.0046	0.3128 ± 0.0035
		Voting (Ensemble)	0.5005 ± 0.0244	0.4517 ± 0.0060	0.4054 ± 0.0024	0.3761 ± 0.0038	**0.3423 ± 0.0026**
		Lasso (Ensemble)	**0.5328 ± 0.0161**	**0.4670 ± 0.0084**	**0.4108 ± 0.0029**	**0.3776 ± 0.0018**	0.3386 ± 0.0015
		Ridge (Ensemble)	0.4305 ± 0.0745	0.4026 ± 0.0515	0.3656 ± 0.0350	0.3417 ± 0.0290	0.3108 ± 0.0255
		ElasticNet (Ensemble)	0.3791 ± 0.1370	0.3493 ± 0.1103	0.3121 ± 0.0933	0.2924 ± 0.0801	0.2696 ± 0.0649
	A549	CIGER (baseline)	0.4812 ± 0.0225	0.4619 ± 0.0085	0.4331 ± 0.0048	0.4100 ± 0.0048	0.3698 ± 0.0042
		RFR	0.5244 ± 0.0122	0.4923 ± 0.0160	0.4573 ± 0.0094	0.4319 ± 0.0071	0.3865 ± 0.0054
		Graph Transformer	0.4785 ± 0.0279	0.4592 ± 0.0133	0.4316 ± 0.0062	0.4092 ± 0.0042	0.3687 ± 0.0030
		SMILES Transformer	0.4477 ± 0.0079	0.4406 ± 0.0087	0.4263 ± 0.0066	0.4053 ± 0.0050	0.3677 ± 0.0042
		SMILES LSTM	0.4805 ± 0.0242	0.4643 ± 0.0091	0.4383 ± 0.0039	0.4147 ± 0.0029	0.3737 ± 0.0025
		Voting (Ensemble)	0.5087 ± 0.0141	0.4833 ± 0.0080	0.4585 ± 0.0070	**0.4360 ± 0.0055**	**0.3878 ± 0.0039**
		Lasso (Ensemble)	**0.5316 ± 0.0126**	**0.4955 ± 0.0118**	**0.4601 ± 0.0079**	0.4341 ± 0.0057	0.3876 ± 0.0044
		Ridge (Ensemble)	0.2561 ± 0.1695	0.2571 ± 0.1594	0.2492 ± 0.1485	0.2429 ± 0.1368	0.2311 ± 0.1127
		ElasticNet (Ensemble)	0.2844 ± 0.1698	0.2683 ± 0.1488	0.2653 ± 0.1368	0.2579 ± 0.1251	0.2424 ± 0.1033
	HA1E	CIGER (baseline)	0.4421 ± 0.0148	0.3980 ± 0.0116	0.3516 ± 0.0071	0.3251 ± 0.0056	0.2959 ± 0.0041
		RFR	0.5234 ± 0.0190	0.4673 ± 0.0109	0.4025 ± 0.0102	0.3649 ± 0.0080	0.3244 ± 0.0057
		Graph Transformer	0.4500 ± 0.0222	0.4086 ± 0.0140	0.3568 ± 0.0105	0.3297 ± 0.0082	0.2994 ± 0.0064
		SMILES Transformer	0.4123 ± 0.0075	0.3787 ± 0.0038	0.3388 ± 0.0034	0.3159 ± 0.0037	0.2900 ± 0.0033
		SMILES LSTM	0.4180 ± 0.0079	0.3759 ± 0.0075	0.3378 ± 0.0081	0.3150 ± 0.0059	0.2897 ± 0.0047
		Voting (Ensemble)	0.4934 ± 0.0250	0.4508 ± 0.0089	0.3967 ± 0.0073	0.3661 ± 0.0063	**0.3287 ± 0.0041**
		Lasso (Ensemble)	**0.5256 ± 0.0182**	**0.4700 ± 0.0115**	**0.4053 ± 0.0088**	**0.3678 ± 0.0074**	0.3258 ± 0.0051
		Ridge (Ensemble)	0.3285 ± 0.1353	0.3078 ± 0.1119	0.2837 ± 0.0899	0.2686 ± 0.0759	0.2508 ± 0.0579
		ElasticNet (Ensemble)	0.4224 ± 0.0481	0.3801 ± 0.0460	0.3380 ± 0.0400	0.3118 ± 0.0346	0.2828 ± 0.0266

Table S3. Performance Metrics of Up-Regulated Gene Expression Ranking Prediction in a Single Cell Line (continue)

Class	Cell line	Model	F1@1	F1@10	F1@50	F1@100	F1@200
Up	HEPG2	CIGER (baseline)	0.4655 ± 0.0185	0.4273 ± 0.0066	0.3817 ± 0.0041	0.3559 ± 0.0046	0.3221 ± 0.0030
		RFR	0.5246 ± 0.0176	0.4731 ± 0.0071	0.4127 ± 0.0049	0.3804 ± 0.0054	0.3396 ± 0.0044
		Graph Transformer	0.4746 ± 0.0158	0.4372 ± 0.0067	0.3848 ± 0.0056	0.3592 ± 0.0040	0.3245 ± 0.0026
		SMILES Transformer	0.4358 ± 0.0193	0.3981 ± 0.0151	0.3584 ± 0.0077	0.3375 ± 0.0057	0.3103 ± 0.0028
		SMILES LSTM	0.4354 ± 0.0068	0.4131 ± 0.0077	0.3693 ± 0.0058	0.3470 ± 0.0038	0.3169 ± 0.0028
		Voting (Ensemble)	0.5121 ± 0.0166	0.4664 ± 0.0086	0.4163 ± 0.0047	**0.3863 ± 0.0046**	**0.3464 ± 0.0030**
		Lasso (Ensemble)	**0.5340 ± 0.0117**	**0.4764 ± 0.0053**	**0.4176 ± 0.0043**	0.3852 ± 0.0049	0.3432 ± 0.0035
		Ridge (Ensemble)	0.3712 ± 0.1706	0.3343 ± 0.1546	0.3013 ± 0.1256	0.2838 ± 0.1098	0.2621 ± 0.0898
		ElasticNet (Ensemble)	0.3240 ± 0.1208	0.3090 ± 0.1021	0.2871 ± 0.0855	0.2745 ± 0.0748	0.2569 ± 0.0598
	HT29	CIGER (baseline)	0.4748 ± 0.0262	0.4518 ± 0.0075	0.4155 ± 0.0041	0.3883 ± 0.0047	0.3540 ± 0.0033
		RFR	**0.5362 ± 0.0219**	**0.4967 ± 0.0057**	**0.4483 ± 0.0038**	**0.4148 ± 0.0039**	0.3724 ± 0.0023
		Graph Transformer	0.4743 ± 0.0201	0.4574 ± 0.0069	0.4179 ± 0.0045	0.3909 ± 0.0040	0.3553 ± 0.0020
		SMILES Transformer	0.4563 ± 0.0107	0.4478 ± 0.0065	0.4132 ± 0.0042	0.3854 ± 0.0037	0.3522 ± 0.0025
		SMILES LSTM	0.4647 ± 0.0180	0.4450 ± 0.0114	0.4114 ± 0.0056	0.3846 ± 0.0042	0.3519 ± 0.0024
		Voting (Ensemble)	0.5148 ± 0.0207	0.4813 ± 0.0081	0.4429 ± 0.0025	0.4134 ± 0.0037	**0.3745 ± 0.0017**
		Lasso (Ensemble)	0.5237 ± 0.0172	0.4956 ± 0.0019	0.4470 ± 0.0018	0.4132 ± 0.0022	0.3713 ± 0.0014
		Ridge (Ensemble)	0.3297 ± 0.1557	0.3162 ± 0.1360	0.2908 ± 0.1244	0.2762 ± 0.1108	0.2591 ± 0.0887
		ElasticNet (Ensemble)	0.1689 ± 0.1534	0.1653 ± 0.1467	0.1605 ± 0.1292	0.1575 ± 0.1168	0.1576 ± 0.1013

Table S4. Performance Metrics of Down-Regulated Gene Expression Ranking Prediction in a Single Cell Line (continue)

Class	Cell line	Model	F1@1	F1@10	F1@50	F1@100	F1@200
Down	MCF7	CIGER (baseline)	0.6122 ± 0.0076	0.5975 ± 0.0140	0.5537 ± 0.0113	0.5158 ± 0.0103	0.4478 ± 0.0073
		RFR	0.6180 ± 0.0086	0.6037 ± 0.0127	0.5603 ± 0.0124	0.5226 ± 0.0105	0.4527 ± 0.0077
		Graph Transformer	0.6120 ± 0.0221	0.5983 ± 0.0132	0.5531 ± 0.0117	0.5157 ± 0.0102	0.4477 ± 0.0073
		SMILES Transformer	0.6020 ± 0.0181	0.5935 ± 0.0157	0.5534 ± 0.0134	0.5176 ± 0.0107	0.4496 ± 0.0082
		SMILES LSTM	0.6045 ± 0.0168	0.5915 ± 0.0163	0.5535 ± 0.0132	0.5170 ± 0.0104	0.4493 ± 0.0072
		Voting (Ensemble)	0.6211 ± 0.0091	0.6049 ± 0.0135	0.5603 ± 0.0128	0.5240 ± 0.0108	**0.4541 ± 0.0077**
		Lasso (Ensemble)	**0.6222 ± 0.0094**	**0.6057 ± 0.0136**	**0.5619 ± 0.0127**	**0.5243 ± 0.0105**	**0.4541 ± 0.0077**
		Ridge (Ensemble)	0.5353 ± 0.0995	0.5221 ± 0.0890	0.4844 ± 0.0816	0.4574 ± 0.0689	0.4016 ± 0.0506
		ElasticNet (Ensemble)	0.4265 ± 0.2310	0.4202 ± 0.2203	0.4021 ± 0.1907	0.3849 ± 0.1627	0.3453 ± 0.1239
	PC3	CIGER (baseline)	0.6375 ± 0.0196	0.6213 ± 0.0135	0.5794 ± 0.0122	0.5387 ± 0.0127	0.4646 ± 0.0094
		RFR	**0.6616 ± 0.0190**	**0.6340 ± 0.0148**	0.5898 ± 0.0136	0.5476 ± 0.0137	0.4713 ± 0.0100
		Graph Transformer	0.6407 ± 0.0143	0.6255 ± 0.0109	0.5792 ± 0.0121	0.5384 ± 0.0124	0.4641 ± 0.0093
		SMILES Transformer	0.6348 ± 0.0069	0.6210 ± 0.0113	0.5800 ± 0.0119	0.5398 ± 0.0129	0.4668 ± 0.0096
		SMILES LSTM	0.6299 ± 0.0173	0.6149 ± 0.0125	0.5801 ± 0.0124	0.5401 ± 0.0141	0.4667 ± 0.0099
		Voting (Ensemble)	0.6551 ± 0.0201	0.6321 ± 0.0121	0.5884 ± 0.0130	0.5465 ± 0.0129	0.4717 ± 0.0095
		Lasso (Ensemble)	0.6582 ± 0.0215	0.6325 ± 0.0136	**0.5900 ± 0.0128**	**0.5478 ± 0.0133**	**0.4718 ± 0.0096**
		Ridge (Ensemble)	0.3860 ± 0.2567	0.3771 ± 0.2464	0.3586 ± 0.2308	0.3354 ± 0.2065	0.3006 ± 0.1609
		ElasticNet (Ensemble)	0.4881 ± 0.2119	0.4780 ± 0.1913	0.4387 ± 0.1709	0.4173 ± 0.1395	0.3736 ± 0.1136
	VCAP	CIGER (baseline)	0.5374 ± 0.0273	0.5471 ± 0.0092	0.5237 ± 0.0071	0.4985 ± 0.0065	0.4498 ± 0.0056
		RFR	**0.5753 ± 0.0314**	0.5625 ± 0.0138	0.5320 ± 0.0073	0.5039 ± 0.0069	0.4536 ± 0.0054
		Graph Transformer	0.5442 ± 0.0163	0.5514 ± 0.0103	0.5242 ± 0.0072	0.4993 ± 0.0060	0.4506 ± 0.0047
		SMILES Transformer	0.5269 ± 0.0288	0.5418 ± 0.0119	0.5238 ± 0.0075	0.4998 ± 0.0060	0.4515 ± 0.0051
		SMILES LSTM	0.5340 ± 0.0257	0.5388 ± 0.0110	0.5231 ± 0.0067	0.4991 ± 0.0071	0.4508 ± 0.0058
		Voting (Ensemble)	0.5569 ± 0.0292	0.5618 ± 0.0108	0.5361 ± 0.0077	0.5102 ± 0.0071	0.4590 ± 0.0057
		Lasso (Ensemble)	0.5750 ± 0.0299	**0.5686 ± 0.0116**	**0.5400 ± 0.0071**	**0.5118 ± 0.0074**	**0.4592 ± 0.0056**
		Ridge (Ensemble)	0.2487 ± 0.2296	0.2548 ± 0.2220	0.2581 ± 0.2081	0.2555 ± 0.1912	0.2408 ± 0.1605
		ElasticNet (Ensemble)	0.3609 ± 0.2119	0.3760 ± 0.2149	0.3758 ± 0.1981	0.3623 ± 0.1837	0.3351 ± 0.1579

Table S4. Performance Metrics of Down-Regulated Gene Expression Ranking Prediction in a Single Cell Line (continue)

Class	Cell line	Model	F1@1	F1@10	F1@50	F1@100	F1@200
Down	A375	CIGER (baseline)	0.4779 ± 0.0179	0.4593 ± 0.0180	0.4222 ± 0.0116	0.3920 ± 0.0100	0.3478 ± 0.0069
		RFR	0.5171 ± 0.0114	0.4906 ± 0.0164	0.4493 ± 0.0112	0.4149 ± 0.0095	0.3650 ± 0.0065
		Graph Transformer	0.4801 ± 0.0231	0.4630 ± 0.0170	0.4249 ± 0.0115	0.3943 ± 0.0102	0.3496 ± 0.0075
		SMILES Transformer	0.4685 ± 0.0194	0.4633 ± 0.0145	0.4280 ± 0.0128	0.3970 ± 0.0119	0.3517 ± 0.0078
		SMILES LSTM	0.4721 ± 0.0168	0.4602 ± 0.0172	0.4281 ± 0.0122	0.3972 ± 0.0110	0.3517 ± 0.0077
		Voting (Ensemble)	0.4970 ± 0.0174	0.4823 ± 0.0160	0.4417 ± 0.0118	0.4087 ± 0.0106	0.3623 ± 0.0074
		Lasso (Ensemble)	**0.5182 ± 0.0097**	**0.4916 ± 0.0154**	**0.4495 ± 0.0118**	**0.4157 ± 0.0101**	**0.3651 ± 0.0071**
		Ridge (Ensemble)	0.4766 ± 0.0459	0.4552 ± 0.0347	0.4216 ± 0.0282	0.3902 ± 0.0270	0.3438 ± 0.0239
		ElasticNet (Ensemble)	0.3624 ± 0.1775	0.3486 ± 0.1532	0.3276 ± 0.1315	0.3124 ± 0.1100	0.2865 ± 0.0827
	A549	CIGER (baseline)	0.3733 ± 0.0109	0.3638 ± 0.0057	0.3457 ± 0.0043	0.3306 ± 0.0027	0.3082 ± 0.0023
		RFR	0.4123 ± 0.0167	0.4006 ± 0.0109	0.3776 ± 0.0066	0.3584 ± 0.0057	**0.3289 ± 0.0044**
		Graph Transformer	0.3565 ± 0.0228	0.3597 ± 0.0179	0.3460 ± 0.0111	0.3306 ± 0.0083	0.3082 ± 0.0056
		SMILES Transformer	0.3490 ± 0.0087	0.3428 ± 0.0052	0.3322 ± 0.0028	0.3195 ± 0.0037	0.2997 ± 0.0025
		SMILES LSTM	0.3582 ± 0.0050	0.3615 ± 0.0111	0.3461 ± 0.0091	0.3300 ± 0.0093	0.3079 ± 0.0066
		Voting (Ensemble)	0.4016 ± 0.0133	0.3847 ± 0.0130	0.3639 ± 0.0063	0.3470 ± 0.0066	0.3229 ± 0.0047
		Lasso (Ensemble)	**0.4195 ± 0.0124**	**0.4035 ± 0.0096**	**0.3785 ± 0.0055**	**0.3590 ± 0.0053**	**0.3289 ± 0.0036**
		Ridge (Ensemble)	0.2158 ± 0.1262	0.2066 ± 0.1177	0.2043 ± 0.1090	0.2020 ± 0.0998	0.2014 ± 0.0834
		ElasticNet (Ensemble)	0.2382 ± 0.1291	0.2404 ± 0.1208	0.2329 ± 0.1058	0.2273 ± 0.0972	0.2180 ± 0.0825
	HA1E	CIGER (baseline)	0.4586 ± 0.0151	0.4408 ± 0.0100	0.4006 ± 0.0083	0.3715 ± 0.0071	0.3294 ± 0.0057
		RFR	0.5239 ± 0.0161	**0.4865 ± 0.0119**	0.4375 ± 0.0124	0.4042 ± 0.0103	**0.3556 ± 0.0079**
		Graph Transformer	0.4682 ± 0.0242	0.4452 ± 0.0117	0.4040 ± 0.0107	0.3745 ± 0.0097	0.3321 ± 0.0078
		SMILES Transformer	0.4397 ± 0.0131	0.4242 ± 0.0112	0.3954 ± 0.0125	0.3701 ± 0.0108	0.3295 ± 0.0080
		SMILES LSTM	0.4266 ± 0.0213	0.4253 ± 0.0164	0.3962 ± 0.0122	0.3704 ± 0.0114	0.3303 ± 0.0088
		Voting (Ensemble)	0.4809 ± 0.0130	0.4625 ± 0.0121	0.4253 ± 0.0124	0.3955 ± 0.0108	0.3505 ± 0.0081
		Lasso (Ensemble)	**0.5254 ± 0.0194**	0.4861 ± 0.0109	**0.4383 ± 0.0125**	**0.4056 ± 0.0105**	0.3553 ± 0.0079
		Ridge (Ensemble)	0.3448 ± 0.1383	0.3383 ± 0.1224	0.3199 ± 0.1066	0.3014 ± 0.0932	0.2736 ± 0.0699
		ElasticNet (Ensemble)	0.4172 ± 0.0732	0.3918 ± 0.0785	0.3622 ± 0.0697	0.3383 ± 0.0594	0.3032 ± 0.0439

Table S4. Performance Metrics of Down-Regulated Gene Expression Ranking Prediction in a Single Cell Line (Continue)

Class	Cell line	Model	F1@1	F1@10	F1@50	F1@100	F1@200
Down	HEPG2	CIGER (baseline)	0.4632 ± 0.0163	0.4427 ± 0.0105	0.4187 ± 0.0070	0.3985 ± 0.0060	0.3593 ± 0.0039
		RFR	0.5017 ± 0.0193	0.4719 ± 0.0152	0.4423 ± 0.0127	0.4188 ± 0.0113	0.3750 ± 0.0083
		Graph Transformer	0.4647 ± 0.0222	0.4473 ± 0.0146	0.4244 ± 0.0089	0.4022 ± 0.0068	0.3622 ± 0.0049
		SMILES Transformer	0.4576 ± 0.0256	0.4330 ± 0.0115	0.4140 ± 0.0103	0.3966 ± 0.0075	0.3585 ± 0.0059
		SMILES LSTM	0.4586 ± 0.0163	0.4413 ± 0.0104	0.4188 ± 0.0096	0.4000 ± 0.0076	0.3604 ± 0.0061
		Voting (Ensemble)	0.4901 ± 0.0108	0.4660 ± 0.0124	0.4374 ± 0.0114	0.4156 ± 0.0089	0.3742 ± 0.0066
		Lasso (Ensemble)	**0.5114 ± 0.0213**	**0.4740 ± 0.0145**	**0.4453 ± 0.0116**	**0.4219 ± 0.0104**	**0.3763 ± 0.0076**
		Ridge (Ensemble)	0.3428 ± 0.1726	0.3330 ± 0.1585	0.3200 ± 0.1425	0.3097 ± 0.1301	0.2881 ± 0.1050
		ElasticNet (Ensemble)	0.3291 ± 0.1055	0.3225 ± 0.1025	0.3125 ± 0.0946	0.2994 ± 0.0861	0.2773 ± 0.0701
	HT29	CIGER (baseline)	0.5324 ± 0.0201	0.5120 ± 0.0076	0.4818 ± 0.0050	0.4535 ± 0.0045	0.4040 ± 0.0037
		RFR	0.5573 ± 0.0128	0.5383 ± 0.0093	0.5024 ± 0.0065	0.4721 ± 0.0050	**0.4183 ± 0.0033**
		Graph Transformer	0.5236 ± 0.0084	0.5152 ± 0.0079	0.4820 ± 0.0045	0.4543 ± 0.0041	0.4048 ± 0.0037
		SMILES Transformer	0.5221 ± 0.0130	0.5118 ± 0.0029	0.4809 ± 0.0050	0.4538 ± 0.0039	0.4050 ± 0.0032
		SMILES LSTM	0.5195 ± 0.0160	0.5138 ± 0.0094	0.4814 ± 0.0032	0.4539 ± 0.0028	0.4046 ± 0.0022
		Voting (Ensemble)	0.5588 ± 0.0117	0.5303 ± 0.0063	0.4955 ± 0.0045	0.4660 ± 0.0029	0.4154 ± 0.0032
		Lasso (Ensemble)	**0.5624 ± 0.0114**	**0.5393 ± 0.0076**	**0.5029 ± 0.0054**	**0.4724 ± 0.0045**	0.4171 ± 0.0034
		Ridge (Ensemble)	0.3291 ± 0.1783	0.3176 ± 0.1662	0.3061 ± 0.1506	0.2924 ± 0.1323	0.2730 ± 0.1055
		ElasticNet (Ensemble)	0.1750 ± 0.1844	0.1743 ± 0.1671	0.1737 ± 0.1552	0.1724 ± 0.1421	0.1709 ± 0.1187

Table S5. Performance Metrics of Up/Down-Regulated Gene Expression Ranking Prediction in a Single Cell Line (continue)

Class	Cell line	Model	F1@1	F1@10	F1@50	F1@100	F1@200
		CIGER (baseline)	0.4674 ± 0.0141	0.4376 ± 0.0113	0.3970 ± 0.0071	0.3694 ± 0.0065	0.3319 ± 0.0046
		RFR	0.5208 ± 0.0133	0.4791 ± 0.0088	0.4282 ± 0.0061	0.3945 ± 0.0049	0.3506 ± 0.0032
		Graph Transformer	0.4617 ± 0.0175	0.4383 ± 0.0100	0.3978 ± 0.0067	0.3704 ± 0.0058	0.3329 ± 0.0046
		SMILES Transformer	0.4562 ± 0.0153	0.4311 ± 0.0083	0.3955 ± 0.0067	0.3681 ± 0.0068	0.3316 ± 0.0051
	A375	SMILES LSTM	0.4599 ± 0.0179	0.4326 ± 0.0107	0.3963 ± 0.0087	0.3690 ± 0.0074	0.3322 ± 0.0054
		Voting (Ensemble)	0.5007 ± 0.0137	0.4684 ± 0.0079	0.4244 ± 0.0060	0.3927 ± 0.0060	**0.3520 ± 0.0043**
		Lasso (Ensemble)	**0.5259 ± 0.0105**	**0.4799 ± 0.0090**	**0.4305 ± 0.0066**	**0.3968 ± 0.0056**	0.3518 ± 0.0039
		Ridge (Ensemble)	0.4552 ± 0.0582	0.4299 ± 0.0419	0.3939 ± 0.0309	0.3660 ± 0.0277	0.3274 ± 0.0244
		ElasticNet (Ensemble)	0.3714 ± 0.1541	0.3498 ± 0.1305	0.3201 ± 0.1121	0.3024 ± 0.0949	0.2780 ± 0.0737
		CIGER (baseline)	0.4281 ± 0.0073	0.4129 ± 0.0059	0.3894 ± 0.0034	0.3703 ± 0.0028	0.3390 ± 0.0025
		RFR	0.4687 ± 0.0109	0.4465 ± 0.0124	0.4175 ± 0.0073	0.3952 ± 0.0058	0.3577 ± 0.0046
		Graph Transformer	0.4178 ± 0.0217	0.4095 ± 0.0148	0.3888 ± 0.0074	0.3699 ± 0.0052	0.3384 ± 0.0032
		SMILES Transformer	0.3986 ± 0.0077	0.3919 ± 0.0062	0.3793 ± 0.0043	0.3624 ± 0.0040	0.3337 ± 0.0033
Up/Down	A549	SMILES LSTM	0.4198 ± 0.0131	0.4130 ± 0.0099	0.3923 ± 0.0063	0.3724 ± 0.0058	0.3408 ± 0.0042
		Voting (Ensemble)	0.4556 ± 0.0134	0.4343 ± 0.0102	0.4109 ± 0.0063	0.3915 ± 0.0056	0.3558 ± 0.0039
		Lasso (Ensemble)	**0.4759 ± 0.0093**	**0.4496 ± 0.0098**	**0.4193 ± 0.0060**	**0.3965 ± 0.0049**	**0.3582 ± 0.0036**
		Ridge (Ensemble)	0.2369 ± 0.1472	0.2321 ± 0.1384	0.2269 ± 0.1286	0.2226 ± 0.1183	0.2163 ± 0.0980
		ElasticNet (Ensemble)	0.2634 ± 0.1449	0.2556 ± 0.1327	0.2492 ± 0.1201	0.2427 ± 0.1103	0.2302 ± 0.0923
		CIGER (baseline)	0.4509 ± 0.0129	0.4198 ± 0.0102	0.3762 ± 0.0069	0.3483 ± 0.0057	0.3127 ± 0.0044
		RFR	0.5241 ± 0.0168	0.4771 ± 0.0108	0.4201 ± 0.0110	0.3845 ± 0.0090	0.3400 ± 0.0067
		Graph Transformer	0.4595 ± 0.0184	0.4272 ± 0.0124	0.3805 ± 0.0101	0.3521 ± 0.0084	0.3157 ± 0.0067
		SMILES Transformer	0.4266 ± 0.0043	0.4019 ± 0.0057	0.3673 ± 0.0073	0.3430 ± 0.0069	0.3097 ± 0.0055
	HA1E	SMILES LSTM	0.4227 ± 0.0101	0.4009 ± 0.0113	0.3671 ± 0.0098	0.3427 ± 0.0085	0.3100 ± 0.0067
		Voting (Ensemble)	0.4877 ± 0.0093	0.4574 ± 0.0102	0.4115 ± 0.0095	0.3809 ± 0.0084	0.3393 ± 0.0060
		Lasso (Ensemble)	**0.5259 ± 0.0171**	**0.4783 ± 0.0106**	**0.4218 ± 0.0103**	**0.3867 ± 0.0087**	**0.3405 ± 0.0064**
		Ridge (Ensemble)	0.3376 ± 0.1358	0.3236 ± 0.1168	0.3020 ± 0.0979	0.2851 ± 0.0843	0.2622 ± 0.0638
		ElasticNet (Ensemble)	0.4215 ± 0.0570	0.3866 ± 0.0599	0.3503 ± 0.0537	0.3251 ± 0.0465	0.2930 ± 0.0349

Table S5. Performance Metrics of Up/Down-Regulated Gene Expression Ranking Prediction in a Single Cell Line (continue)

Class	Cell line	Model	F1@1	F1@10	F1@50	F1@100	F1@200
Up/Down	HEPG2	CIGER (baseline)	0.4647 ± 0.0130	0.4354 ± 0.0082	0.4004 ± 0.0051	0.3773 ± 0.0049	0.3407 ± 0.0032
		RFR	0.5139 ± 0.0149	0.4729 ± 0.0103	0.4276 ± 0.0087	0.3997 ± 0.0080	0.3573 ± 0.0061
		Graph Transformer	0.4701 ± 0.0117	0.4425 ± 0.0088	0.4048 ± 0.0061	0.3807 ± 0.0046	0.3434 ± 0.0032
		SMILES Transformer	0.4472 ± 0.0177	0.4161 ± 0.0130	0.3865 ± 0.0086	0.3671 ± 0.0064	0.3344 ± 0.0042
		SMILES LSTM	0.4485 ± 0.0098	0.4278 ± 0.0075	0.3943 ± 0.0070	0.3735 ± 0.0052	0.3386 ± 0.0039
		Voting (Ensemble)	0.5025 ± 0.0092	0.4678 ± 0.0101	0.4275 ± 0.0075	0.4011 ± 0.0061	**0.3600 ± 0.0040**
		Lasso (Ensemble)	**0.5236 ± 0.0103**	**0.4758 ± 0.0096**	**0.4316 ± 0.0077**	**0.4036 ± 0.0072**	0.3597 ± 0.0052
		Ridge (Ensemble)	0.3571 ± 0.1712	0.3341 ± 0.1564	0.3108 ± 0.1338	0.2968 ± 0.1197	0.2751 ± 0.0973
		ElasticNet (Ensemble)	0.3282 ± 0.1124	0.3168 ± 0.1021	0.3001 ± 0.0900	0.2870 ± 0.0804	0.2671 ± 0.0650
	HT29	CIGER (baseline)	0.5070 ± 0.0157	0.4822 ± 0.0074	0.4487 ± 0.0044	0.4210 ± 0.0046	0.3790 ± 0.0035
		RFR	**0.5473 ± 0.0169**	**0.5177 ± 0.0069**	**0.4754 ± 0.0044**	**0.4434 ± 0.0040**	**0.3954 ± 0.0024**
		Graph Transformer	0.4997 ± 0.0061	0.4865 ± 0.0064	0.4500 ± 0.0042	0.4227 ± 0.0039	0.3800 ± 0.0027
		SMILES Transformer	0.4905 ± 0.0072	0.4802 ± 0.0042	0.4471 ± 0.0044	0.4196 ± 0.0035	0.3786 ± 0.0027
		SMILES LSTM	0.4925 ± 0.0104	0.4797 ± 0.0095	0.4464 ± 0.0039	0.4193 ± 0.0031	0.3783 ± 0.0021
		Voting (Ensemble)	0.5414 ± 0.0183	0.5065 ± 0.0057	0.4695 ± 0.0030	0.4398 ± 0.0030	0.3945 ± 0.0022
		Lasso (Ensemble)	0.5435 ± 0.0131	**0.5177 ± 0.0043**	0.4750 ± 0.0032	0.4428 ± 0.0033	0.3942 ± 0.0023
		Ridge (Ensemble)	0.3297 ± 0.1652	0.3179 ± 0.1497	0.2987 ± 0.1368	0.2844 ± 0.1211	0.2661 ± 0.0970
		ElasticNet (Ensemble)	0.1721 ± 0.1688	0.1700 ± 0.1568	0.1672 ± 0.1421	0.1650 ± 0.1293	0.1643 ± 0.1099

Table S6. Performance Metrics of Up-Regulated Gene Expression Ranking Prediction in Multiple Cell Lines

Class	Cell line	Model	F1@1	F1@10	F1@50	F1@100	F1@200
Up	Three cell lines (MCF7, PC3, VCAP)	CIGER (baseline)	0.5777 ± 0.0089	0.5383 ± 0.0069	0.4790 ± 0.0052	0.4408 ± 0.0041	0.3924 ± 0.0030
		RFR	0.5950 ± 0.0086	0.5522 ± 0.0034	0.4909 ± 0.0032	0.4506 ± 0.0024	0.3990 ± 0.0018
		Graph Transformer	0.5833 ± 0.0112	0.5398 ± 0.0045	0.4800 ± 0.0046	0.4417 ± 0.0037	0.3930 ± 0.0029
		SMILES Transformer	0.5530 ± 0.0127	0.5157 ± 0.0063	0.4656 ± 0.0046	0.4323 ± 0.0035	0.3879 ± 0.0029
		SMILES LSTM	0.5511 ± 0.0171	0.5138 ± 0.0062	0.4655 ± 0.0042	0.4316 ± 0.0032	0.3870 ± 0.0030
		Voting (Ensemble)	**0.6012 ± 0.0084**	**0.5567 ± 0.0027**	**0.4995 ± 0.0028**	**0.4605 ± 0.0023**	**0.4084 ± 0.0014**
		Lasso (Ensemble)	0.5990 ± 0.0068	0.5523 ± 0.0022	0.4919 ± 0.0036	0.4522 ± 0.0028	0.4008 ± 0.0019
		Ridge (Ensemble)	0.1995 ± 0.2084	0.1935 ± 0.1946	0.1830 ± 0.1708	0.1764 ± 0.1500	0.1714 ± 0.1229
		ElasticNet (Ensemble)	0.3203 ± 0.2355	0.3023 ± 0.2168	0.2862 ± 0.1944	0.2720 ± 0.1739	0.2513 ± 0.1447
	Eight cell lines	CIGER (baseline)	0.5430 ± 0.0066	0.4967 ± 0.0040	0.4385 ± 0.0028	0.4039 ± 0.0023	0.3602 ± 0.0017
		RFR	0.5567 ± 0.0057	0.5077 ± 0.0026	0.4503 ± 0.0017	0.4147 ± 0.0014	0.3697 ± 0.0011
		Graph Transformer	0.5471 ± 0.0029	0.4994 ± 0.0040	0.4408 ± 0.0027	0.4054 ± 0.0022	0.3614 ± 0.0012
		SMILES Transformer	0.4893 ± 0.0067	0.4581 ± 0.0030	0.4155 ± 0.0017	0.3877 ± 0.0015	0.3508 ± 0.0012
		SMILES LSTM	0.4814 ± 0.0067	0.4513 ± 0.0034	0.4126 ± 0.0022	0.3854 ± 0.0020	0.3491 ± 0.0013
		Voting (Ensemble)	**0.5768 ± 0.0060**	**0.5276 ± 0.0041**	**0.4693 ± 0.0026**	**0.4323 ± 0.0022**	**0.3832 ± 0.0012**
		Lasso (Ensemble)	0.5735 ± 0.0050	0.5198 ± 0.0036	0.4589 ± 0.0022	0.4216 ± 0.0015	0.3739 ± 0.0010
		Ridge (Ensemble)	0.3888 ± 0.1881	0.3653 ± 0.1677	0.3335 ± 0.1425	0.3118 ± 0.1267	0.2823 ± 0.1047
		ElasticNet (Ensemble)	0.3876 ± 0.1732	0.3619 ± 0.1581	0.3291 ± 0.1359	0.3076 ± 0.1210	0.2782 ± 0.0996

Table S7. Performance Metrics of Down-Regulated Gene Expression Ranking Prediction in Multiple Cell Lines

Class	Cell line	Model	F1@1	F1@10	F1@50	F1@100	F1@200
Down	Three cell lines (MCF7, PC3, VCAP)	CIGER (baseline)	0.6009 ± 0.0094	0.5878 ± 0.0083	0.5512 ± 0.0060	0.5175 ± 0.0053	0.4542 ± 0.0039
		RFR	**0.6150 ± 0.0106**	0.5969 ± 0.0056	0.5587 ± 0.0048	0.5236 ± 0.0044	0.4591 ± 0.0031
		Graph Transformer	0.5976 ± 0.0150	0.5877 ± 0.0074	0.5510 ± 0.0056	0.5171 ± 0.0056	0.4544 ± 0.0039
		SMILES Transformer	0.5886 ± 0.0089	0.5819 ± 0.0078	0.5507 ± 0.0068	0.5180 ± 0.0057	0.4557 ± 0.0041
		SMILES LSTM	0.5880 ± 0.0056	0.5825 ± 0.0072	0.5501 ± 0.0062	0.5181 ± 0.0053	0.4555 ± 0.0041
		Voting (Ensemble)	0.6049 ± 0.0109	0.5959 ± 0.0063	0.5595 ± 0.0056	0.5256 ± 0.0051	0.4611 ± 0.0035
		Lasso (Ensemble)	0.6134 ± 0.0096	**0.5985 ± 0.0058**	**0.5612 ± 0.0053**	**0.5264 ± 0.0049**	**0.4612 ± 0.0034**
		Ridge (Ensemble)	0.2092 ± 0.2036	0.1997 ± 0.1856	0.1979 ± 0.1710	0.1890 ± 0.1491	0.1903 ± 0.1286
		ElasticNet (Ensemble)	0.3567 ± 0.2420	0.3566 ± 0.2404	0.3437 ± 0.2242	0.3259 ± 0.2036	0.2912 ± 0.1649
	Eight cell lines	CIGER (baseline)	0.5200 ± 0.0065	0.5010 ± 0.0059	0.4668 ± 0.0045	0.4380 ± 0.0038	0.3893 ± 0.0025
		RFR	0.5438 ± 0.0056	0.5208 ± 0.0042	0.4842 ± 0.0027	0.4533 ± 0.0025	0.4012 ± 0.0016
		Graph Transformer	0.5225 ± 0.0065	0.5040 ± 0.0034	0.4689 ± 0.0036	0.4401 ± 0.0030	0.3911 ± 0.0018
		SMILES Transformer	0.4995 ± 0.0063	0.4900 ± 0.0038	0.4616 ± 0.0032	0.4346 ± 0.0025	0.3872 ± 0.0021
		SMILES LSTM	0.5033 ± 0.0082	0.4901 ± 0.0056	0.4621 ± 0.0040	0.4352 ± 0.0036	0.3874 ± 0.0024
		Voting (Ensemble)	0.5468 ± 0.0017	0.5238 ± 0.0035	0.4861 ± 0.0030	0.4549 ± 0.0027	**0.4033 ± 0.0019**
		Lasso (Ensemble)	**0.5499 ± 0.0045**	**0.5261 ± 0.0050**	**0.4885 ± 0.0033**	**0.4568 ± 0.0029**	**0.4033 ± 0.0019**
		Ridge (Ensemble)	0.3779 ± 0.1841	0.3727 ± 0.1713	0.3548 ± 0.1519	0.3357 ± 0.1370	0.3023 ± 0.1117
		ElasticNet (Ensemble)	0.3737 ± 0.1747	0.3645 ± 0.1676	0.3472 ± 0.1494	0.3294 ± 0.1340	0.2960 ± 0.1081

Table S8. AUROC of Up/Down-Regulated Gene Expression Ranking Prediction in Single and Multiple Cell Lines (continue)

Class	Model	MCF7	PC3	VCAP	A549	A375
Up/Down	CIGER (baseline)	0.6624 ± 0.0025	0.6672 ± 0.0054	0.6895 ± 0.0033	0.6267 ± 0.0030	0.6060 ± 0.0024
	RFR	0.6658 ± 0.0028	0.6722 ± 0.0056	0.6954 ± 0.0030	0.6416 ± 0.0043	0.6209 ± 0.0017
	Graph Transformer	0.6614 ± 0.0030	0.6666 ± 0.0046	0.6892 ± 0.0025	0.6264 ± 0.0027	0.6054 ± 0.0012
	SMILES Transformer	0.6585 ± 0.0027	0.6653 ± 0.0051	0.6854 ± 0.0030	0.6212 ± 0.0028	0.6022 ± 0.0027
	SMILES LSTM	0.6586 ± 0.0025	0.6652 ± 0.0053	0.6852 ± 0.0032	0.6200 ± 0.0025	0.6026 ± 0.0037
	Voting (Ensemble)	0.6695 ± 0.0026	0.6762 ± 0.0058	0.6997 ± 0.0032	0.6369 ± 0.0037	0.6182 ± 0.0028
	Lasso (Ensemble)	0.6666 ± 0.0025	0.6725 ± 0.0055	0.6970 ± 0.0032	0.6417 ± 0.0039	0.6208 ± 0.0020
	Ridge (Ensemble)	0.4928 ± 0.1450	0.5948 ± 0.1359	0.5061 ± 0.1334	0.4889 ± 0.1096	0.5744 ± 0.0454
	ElasticNet (Ensemble)	0.5450 ± 0.1353	0.4798 ± 0.1474	0.6369 ± 0.0416	0.5867 ± 0.0669	0.5333 ± 0.0920

Table S8. AUROC of Up/Down-Regulated Gene Expression Ranking Prediction in Single and Multiple Cell Lines (continue)

Class	Model	HA1E	HEPG2	HT29	Three Cell Lines	Eight Cell Lines
Up/Down	CIGER (baseline)	0.5867 ± 0.0045	0.6073 ± 0.0041	0.6352 ± 0.0034	0.6744 ± 0.0018	0.6388 ± 0.0011
	RFR	0.6079 ± 0.0044	0.6219 ± 0.0042	0.6501 ± 0.0014	0.6788 ± 0.0011	0.6471 ± 0.0007
	Graph Transformer	0.5854 ± 0.0052	0.6098 ± 0.0021	0.6369 ± 0.0021	0.6747 ± 0.0018	0.6399 ± 0.0012
	SMILES Transformer	0.5776 ± 0.0034	0.5994 ± 0.0029	0.6327 ± 0.0031	0.6719 ± 0.0017	0.6316 ± 0.0010
	SMILES LSTM	0.5753 ± 0.0033	0.5987 ± 0.0029	0.6335 ± 0.0031	0.6710 ± 0.0019	0.6340 ± 0.0017
	Voting (Ensemble)	0.5997 ± 0.0034	0.6204 ± 0.0033	0.6455 ± 0.0025	0.6831 ± 0.0013	0.6519 ± 0.0013
	Lasso (Ensemble)	0.6070 ± 0.0044	0.6233 ± 0.0037	0.6477 ± 0.0022	0.6802 ± 0.0013	0.6494 ± 0.0008
	Ridge (Ensemble)	0.5512 ± 0.0750	0.5471 ± 0.0695	0.4646 ± 0.1183	0.5517 ± 0.1070	0.5570 ± 0.1036
	ElasticNet (Ensemble)	0.5186 ± 0.0964	0.5710 ± 0.0702	0.4972 ± 0.1167	0.4994 ± 0.1348	0.5383 ± 0.0918

Table S9. Optimal Hyperparameters of QSAR Models for PARP1

Fingerprints	Model	Optimal Parameter
MACCS/ECFP6	LGBM	{'boosting_type': 'dart', 'learning_rate': 0.01, 'n_estimators': 2500}
	RFC	{'max_depth': 10, 'n_estimators': 3000}
	XGB	{'booster': 'gbtree', 'learning_rate': 0.01, 'max_depth': 10, 'n_estimators': 1500}
MACCS/FCFP4	LGBM	{'boosting_type': 'dart', 'learning_rate': 0.05, 'n_estimators': 500}
	RFC	{'max_depth': 20, 'n_estimators': 2000}
	XGB	{'booster': 'gbtree', 'learning_rate': 0.01, 'max_depth': 10, 'n_estimators': 1500}
MACCS/MFBERT	LGBM	{'boosting_type': 'dart', 'learning_rate': 0.05, 'n_estimators': 1000}
	RFC	{'max_depth': 10, 'n_estimators': 50}
	XGB	{'booster': 'gbtree', 'learning_rate': 0.1, 'max_depth': 10, 'n_estimators': 3000}
MACCS/PCFP	LGBM	{'boosting_type': 'dart', 'learning_rate': 0.01, 'n_estimators': 2500}
	RFC	{'max_depth': 20, 'n_estimators': 2500}
	XGB	{'booster': 'gbtree', 'learning_rate': 0.05, 'max_depth': 20, 'n_estimators': 1500}

Table S10. Performance Metrics of QSAR Models for PARP1

Fingerprints	Model	Train Accuracy	Train Precision	Train Recall	Train F1-score	Train MCC	Test Accuracy	Test Precision	Test Recall	Test F1-score	Test MCC
MACCS/ECFP6	LGBM	0.995	0.945	1.000	0.972	0.969	0.992	0.927	0.986	0.956	0.952
	RFC	0.989	0.891	0.995	0.940	0.936	0.989	0.900	0.979	0.938	0.932
	XGB	1.000	1.000	1.000	1.000	1.000	0.992	0.954	0.945	0.950	0.945
MACCS/FCFP4	LGBM	0.993	0.918	1.000	0.957	0.954	0.991	0.915	0.984	0.948	0.944
	RFC	0.996	0.954	1.000	0.977	0.975	0.992	0.939	0.972	0.955	0.951
	XGB	0.999	0.995	0.996	0.996	0.995	0.992	0.952	0.951	0.952	0.947
MACCS/MFBERT	LGBM	1.000	1.000	1.000	1.000	1.000	0.990	0.921	0.970	0.945	0.940
	RFC	0.991	0.900	1.000	0.947	0.944	0.985	0.897	0.926	0.912	0.903
	XGB	1.000	1.000	1.000	1.000	1.000	0.989	0.949	0.924	0.937	0.931
MACCS/PCFP	LGBM	0.993	0.923	1.000	0.960	0.957	0.989	0.897	0.988	0.940	0.936
	RFC	0.996	0.952	1.000	0.975	0.973	0.990	0.928	0.959	0.944	0.939
	XGB	1.000	0.999	0.999	0.999	0.999	0.991	0.952	0.945	0.949	0.944
